# Advances in emergency medicine research in India: abstracts from the EMCON 2014 international conference

**DOI:** 10.1186/1865-1380-8-S1-I1

**Published:** 2015-04-22

**Authors:** Bobby Kapur, Sanjay Mehta, Kedar Toraskar, Kumar Alagappan

**Affiliations:** 1 Baylor College of Medicine, Houston, TX, USA; 2 Kokilaben Dhirubhai Ambani Hospital & Medical Research Institute, Mumbai, India; 3 New Age Wolkhardt Hospital, Mumbai, India

## 

Within India, Emergency Medicine (EM) is a rapidly evolving and expanding specialty. EM has received increased recognition by the national government the past few years with the accreditation of training positions through the Medical Council of India and the National Board of Examinations. These recent advances have been gained through more than a decade of efforts by the Society for Emergency Medicine, India (SEMI) along with partner organizations such as the International Federation for Emergency Medicine (IFEM), the American Academy for Emergency Medicine in India (AAEMI) and the Global Academy of Emergency Medicine (GAEM).

SEMI sponsored India’s premier Emergency Medicine conference, EMCON 2014, in Mumbai from November 6-9, 2014. This meeting attracted over 1,200 delegates both nationally and internationally. The importance of this meeting was highlighted by the advancement of clinical research now being conducted by EM physicians across India.

Both AAEMI and GAEM sponsored the research section at EMCON 2014. Over 170 abstracts were submitted, and fourteen of the top papers from the conference are presented as a supplement in this journal. As the specialty develops, new concerns are being raised and brought to the forefront. Many of these issues are widespread to emergency medicine globally, but many are also matters that are endemic to India and can now be addressed.

## Pre-Hospital Care and Emergency Medical Services (EMS)

The specialty of EM is expanding beyond the borders of the EDs in India into the pre-hospital care setting. Emergency physicians are becoming more involved in the planning and protocols involved in the transport of critically ill patients by medical providers. The abstract by Bharati, Taraphdhar and Choudhary shows the need for more regional centralization of pre-hospital services to provide better care for patients. Taraphdhar and Reddy present the efficacy of using Isoprenaline for patients in complete heart block who are transported by ambulance.

## Trauma and Road Traffic Injuries

As the number of cars increases exponentially, India is facing the enormous burden of large numbers of Road Traffic Injuries (RTIs). Abstracts by Radha and by Ahuja, *et. al* and by Dilip, *et. al*, focus on the impact of RTIs and the challenges Indian EDs face managing these trauma patients. Shankar and Mohanasundaram compare the effectiveness of Ketamine and Propofol versus Fentanyl and Propofol as induction agents for rapid sequence endotracheal intubations.

## Mass gathering events

India has a long history of large public events from political rallies to religious festivals, but sporting events are bringing a new complexity with civilians being active participants in events such as marathons. Fatima, *et. al*., provide one of the first ever analysis of medical injuries of runners in a marathon in India.

## Communicable diseases and sepsis

Indian Emergency Physicians have experience treating malaria, TB, and respiratory diseases, but the abundant use of antibiotics that are readily available without prescription in many pharmacies has led to an alarming increase of antibiotic associated diarrhea. Shah et al, document the effect of *Clostridium difficile* diarrhea in patients presenting to the ED. In addition, early treatment of patients with sepsis requires rapid identification of severe disease and potential markers to predict outcomes. Lahiri and Chandresekaran present a novel use of Red Cell Distribution Width (RDW) as a marker for mortality in sepsis patients. Sivaraj, Tausif, and Parivalavan provide the results from their validation study of a “Fever Protocol” to reduce antibiotic usage and hospital admissions for patients with undifferentiated fever.

## Non-communicable diseases

With India’s economic development, the epidemiology of patients presenting to EDs is shifting from communicable to non-communicable diseases such as diabetes, asthma, and hypertension. Diagnosis of acute cardiac causes for chest pain is a global challenge for all Emergency Physicians. Natarajan *et al* presents the “HEART Score” as a clinical decision tool that Emergency Physicians may use to help risk stratify patients with acute chest pain. Sharma *et al* describe the difficulty of identifying Myocardial Infarctions in patients less than 40 years of age.

## Emergency department operations

Since India has the world’s second largest population at over 1.2 billion people, ED’s in India tend to be high-volume, high-acuity areas of healthcare delivery. Because of its complex environment, providing safe and high-quality patient ED care remains an ongoing challenge. Mallick *et al* present a prospective study that evaluates the sensitivity of a clinical decision tool for the safe discharge of patients from the ED.

EM research in India is now being conducted in a more systematic way and within academic Emergency Departments (EDs). Of the 14 papers presented here, most of the research studies were done at institutions that train EM physicians. This finding once again reiterates the need for the expansion of academic departments and training programs in India to meet the enormous demand for high-quality emergency care within the country.

